# A Common Neural Account for Social and Nonsocial Decisions

**DOI:** 10.1523/JNEUROSCI.0375-22.2022

**Published:** 2022-11-30

**Authors:** Desislava H. Arabadzhiyska, Oliver G.B. Garrod, Elsa Fouragnan, Emanuele De Luca, Philippe G. Schyns, Marios G. Philiastides

**Affiliations:** ^1^School of Psychology and Neuroscience, University of Glasgow, Glasgow G12 8QB, United Kingdom; ^2^Centre for Cognitive Neuroimaging, University of Glasgow, Glasgow G12 8QB, United Kingdom; ^3^School of Psychology, University of Plymouth, Plymouth PL4 8AA, United Kingdom

**Keywords:** decision making, EEG, evidence accumulation, fMRI, modeling

## Abstract

To date, social and nonsocial decisions have been studied largely in isolation. Consequently, the extent to which social and nonsocial forms of decision uncertainty are integrated using shared neurocomputational resources remains elusive. Here, we address this question using simultaneous electroencephalography (EEG)-functional magnetic resonance imaging (fMRI) in healthy human participants (young adults of both sexes) and a task in which decision evidence in social and nonsocial contexts varies along comparable scales. First, we identify time-resolved build-up of activity in the EEG, akin to a process of evidence accumulation (EA), across both contexts. We then use the endogenous trial-by-trial variability in the slopes of these accumulating signals to construct parametric fMRI predictors. We show that a region of the posterior-medial frontal cortex (pMFC) uniquely explains trial-wise variability in the process of evidence accumulation in both social and nonsocial contexts. We further demonstrate a task-dependent coupling between the pMFC and regions of the human valuation system in dorso-medial and ventro-medial prefrontal cortex across both contexts. Finally, we report domain-specific representations in regions known to encode the early decision evidence for each context. These results are suggestive of a domain-general decision-making architecture, whereupon domain-specific information is likely converted into a “common currency” in medial prefrontal cortex and accumulated for the decision in the pMFC.

**SIGNIFICANCE STATEMENT** Little work has directly compared social-versus-nonsocial decisions to investigate whether they share common neurocomputational origins. Here, using combined electroencephalography (EEG)-functional magnetic resonance imaging (fMRI) and computational modeling, we offer a detailed spatiotemporal account of the neural underpinnings of social and nonsocial decisions. Specifically, we identify a comparable mechanism of temporal evidence integration driving both decisions and localize this integration process in posterior-medial frontal cortex (pMFC). We further demonstrate task-dependent coupling between the pMFC and regions of the human valuation system across both contexts. Finally, we report domain-specific representations in regions encoding the early, domain-specific, decision evidence. These results suggest a domain-general decision-making architecture, whereupon domain-specific information is converted into a common representation in the valuation system and integrated for the decision in the pMFC.

## Introduction

Most strategic decisions occur under considerable uncertainty. For example, when investing in the stock market, a trader may use only purely probabilistic models to estimate risk in the market's fluctuations. In contrast, when negotiating a deal in person, the trader's risk assessment may rely instead on how trustworthy the other party appears (Fouragnan et al., 2013; Griessinger and Coricelli, 2015). Similarly, the decision to undergo a risky surgical operation may depend on online statistics regarding past success rates or seek the advice of a trustworthy friend who has recently undergone a similar operation.

In standard economic utility models (Morgenstern and Von Neumann, 1953), the rules governing such decisions are the same, regardless of whether the source of uncertainty is social or nonsocial in nature (e.g., a human or an online platform). In correspondence with this view, it has been argued that the same neural network processes the different forms of uncertainty and converts the values associated with different choice alternatives into a “common currency” (Ruff and Fehr, 2014). This view has gathered support over the years with some studies comparing the learning mechanisms employed across social and nonsocial decisions (Tarantola et al., 2017) and implicating prefrontal structures in the processing of both social and nonsocial value (L.T. Harris et al., 2007; Konovalov et al., 2021). Nonetheless, these examinations focused mainly on one subtype of social choices, based on self-versus-other considerations (A. Harris et al., 2018), and with a special emphasis on value computation during the learning stages of decision-making (Behrens et al., 2008; Tarantola et al., 2017).

While these studies add valuable insight into the extent of shared value representations across social and nonsocial contexts, the domain-generality hypothesis has not been fully established, with some studies providing evidence in support of largely dedicated brain networks for encoding social and nonsocial forms of decision uncertainty and for assigning value to different choice alternatives (Behrens et al., 2008; Tarantola et al., 2017; A. Harris et al., 2018; Ugazio et al., 2022). Moreover, previous attempts to compare social and nonsocial decisions fall short of equalizing the different forms of decision uncertainty and often collect the data across social and nonsocial contexts in different subjects/experiments (Krajbich et al., 2015; Tarantola et al., 2017). Importantly, none of the previous comparisons have attempted to investigate potential implementational commonalities of how the relevant evidence is integrated for the decision [i.e., domain generality of the process of evidence accumulation (EA)].

We argue that these are important considerations in providing a unified framework for integrating social and nonsocial decisions. Correspondingly, to characterize the full cascade of constituent neural processes both the algorithmic (i.e., what mechanistic rules are involved) and implementational (i.e., which brain regions are involved) levels of social and nonsocial choices need to be considered. This approach, in turn, will offer an opportunity to further ascertain whether the distinction between social and nonsocial choices in the brain is categorical or could be better understood in terms of a shared relationship (Lockwood et al., 2020).

To address these issues, here, we design a novel task in which decision evidence in social and nonsocial contexts varies along comparable scales. Importantly, in our task, the social information is carried by the social interpretation of facial features (i.e., a social cue) rather than self-versus-other or group decisions (Suzuki et al., 2015; Park et al., 2019). Across contexts, we test whether there is a common embedding of decision evidence as well as a common mechanism for integrating this evidence using simultaneous electroencephalography (EEG) and functional magnetic resonance imaging (fMRI; henceforth EEG-fMRI) in an attempt to expand the scope of the typical “common currency” investigation.

In doing so, we identify, in both contexts, centroparietal EEG signals exhibiting decision dynamics consistent with a common process of EA. Consistent with such domain-general mechanisms, the trial-by-trial temporal variability in these accumulating signals is reflected in the fMRI data in the region of the posterior-medial frontal cortex (pMFC) previously implicated in other types of decisions (Pisauro et al., 2017). Moreover we report a trial-wise and task-dependent modulation of the pMFC with established regions of the human valuation system in the medial prefrontal cortex (Chib et al., 2009; Philiastides et al., 2010; Clithero and Rangel, 2014), suggestive of decision evidence embedded within a “common currency” space. Finally, we also identify domain-specific activations known to reflect the early encoding of the relevant social and nonsocial decision evidence.

## Materials and Methods

### Participants

Forty participants were recruited through The University of Glasgow subject pool. Since facial perception may depend on one's race and racial history (Scott and Monesson, 2009), participants were chosen to be whites, aged 18–35 to match the available face stimuli (see below). Two participants were removed because of poor behavior (one had near chance performance across all levels of reward probability in the social context whereas the other had chosen to nearly always “Play” across all levels of reward probability in the nonsocial context) and seven participants because of noisy EEG signals in the scanner leading to poor (chance) discrimination performance. The remaining 31 subjects (12 males, 19 females), were included in all subsequent analyses. They all had normal or corrected-to-normal vision and reported no history of psychiatric, neurologic or major medical problems, and were free of psychoactive medications at the time of the study. The study was approved by the College of Science and Engineering Ethics Committee at the University of Glasgow (300180147) and informed consent was obtained from all participants.

Our original sample size was based on previous studies employing similar behavioral decision tasks and neuroimaging methods (Philiastides et al., 2014; Pisauro et al., 2017; Gherman and Philiastides, 2018). We further ran a power analysis (G*Power software; Faul et al., 2007) using the behavioral data from Pisauro et al. (2017; i.e., one sample two-tailed *t* test on β coefficients from a single-trial regression analysis to examine the effects of task difficulty on choice [reaction time (RT) data] with an α level of 0.05, power of 0.95 to inform our estimated effect size (0.847). This power analysis indicated a minimum sample size of 21 participants. We effectively doubled this estimate (*N* = 40) to account for the potential removal of participants on the basis of poor behavioral performance and noisy neuroimaging data, given the added challenges of the simultaneous EEG-fMRI acquisition. Our eventual sample size of 31 participants is comparable to previous EEG-fMRI studies in which reliable brain differences were observed in the EEG/fMRI data (Fouragnan et al., 2015, 2017; Pisauro et al., 2017; Gherman and Philiastides, 2018).

### Stimuli

**Figure 1.**
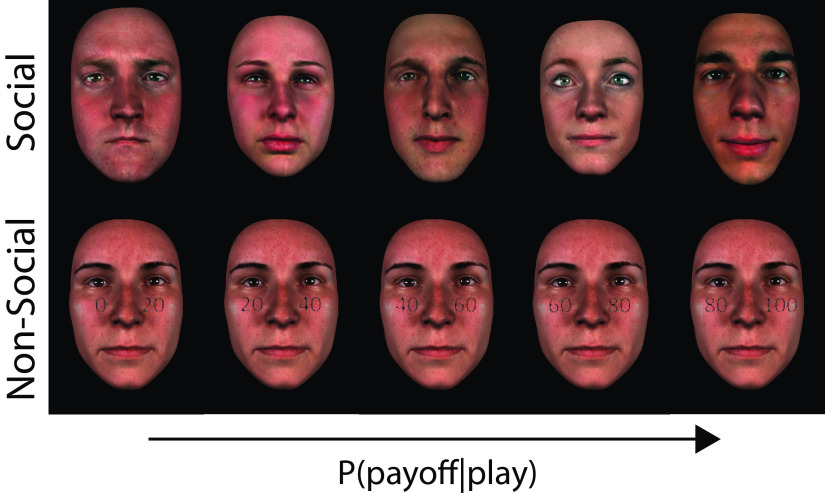
Sample stimuli from a representative participant. Top, Social stimuli at five different (participant–specific) indirect trustworthiness levels, matching the pure reward probability levels used for the nonsocial stimuli. For each participant there were, on average, 28 unique face identities in each of the five reward probability levels. Bottom, Nonsocial stimuli with five explicit reward probability levels (given a “Play” choice) superimposed on a face neutral for trustworthiness (i.e., 0.5 reward probability). The same neutral face was used for all trustworthiness levels and across participants. Photo-realistic face images were obtained using the procedure described previously (Gill et al., 2014) and summarized in Materials and Methods.

We used a set of 150 photorealistic face images (400 × 300 pixels). We presented all stimuli centrally via an LCD projector (frame rate = 60 Hz) on a screen placed at the rear opening of the bore of the MRI scanner, and viewed through a mirror mounted on the head coil (distance to screen = 95 cm), using Presentation software (Neurobehavioral Systems Inc.). These face images were assigned to the five levels of reward probabilities given a “Play” choice [P(payoff|play)={0−0.2,0.2−0.4,0.4−0.6,0.6−0.8,0.8−1}] used in the main task (Fig. 1), based on participant-specific indirect trustworthiness ratings for each face (see procedure below). Nonetheless, to encourage a broad range of indirect trustworthiness reports from our participants, we manipulated these images to obtain versions across a potentially wide range of trustworthiness levels.

Specifically, we used a reverse correlation procedure (Ahumada and Lovell, 1971) to first identify features associated with higher trustworthiness scores and we then manipulated these features in all faces to create different trustworthiness versions of each face using a Generative Face Grammar (Yu et al., 2012). We estimated the facial features associated with trustworthiness judgements from a separate set of 416 faces that were each first 3D-captured using a Di4D (Dimensional Imaging) facial capture system and subsequently rendered and rated for their trustworthiness by 49 independent observers, based on a reverse-correlation procedure reported previously (Zhan et al., 2019).

Each captured face is described by a (4735*3 × 1) vector of 3D mesh vertex coordinates (*x*,*y*,*z*) and a (800*600*3 × 1) vector of texture pixel colors (r,g,b). For each mesh vertex coordinate and texture pixel we fit a multiple linear regression model predicting the value of the coordinate or pixel as a function of the following predictors: average trustworthiness rating over the 49 observers, age of the face, sex of the face, ethnicity of the face, plus a constant term and interaction terms between the predictors, yielding a set of linear coefficients for each vertex coordinate and pixel. Categorical predictors were coded as one-hot vectors, continuous predictors were coded as their real values. Each individual face vertex and texture pixel is then described by the output of this linear model at the given values of the predictors plus an identity-specific residual term.

To generate individual faces of varying degrees of perceived trustworthiness for a specific identity we evaluated the linear model at varying levels of the trustworthiness predictor while holding the remaining predictors constant (at the observed values for this identity) and finally added the identity-specific residual term and rendered the resulting 3D model. This procedure was first applied to 131 identities, made up of 61 male images, 70 female images (all white), and an additional set of 19 new identities were included (to increase the image sample size to 150) by perturbing the identity-specific residuals in the above procedure with noise in the directions of the principal components of the identity space.

Using this procedure, we created twenty trustworthiness versions for each face, 1 representing the least trustworthy version and 20 displaying a trustworthy version of the face. Only one version per face identity (original and fabricated) was chosen for the main experiment. As noted above, this procedure was adopted purely for increasing the likelihood that stimuli would fall into a wide range of different trustworthiness categories, though ultimately the final categorization of each face was based on the participant-specific reports as we explain in more detail below.

### Experimental paradigm

**Figure 2.**
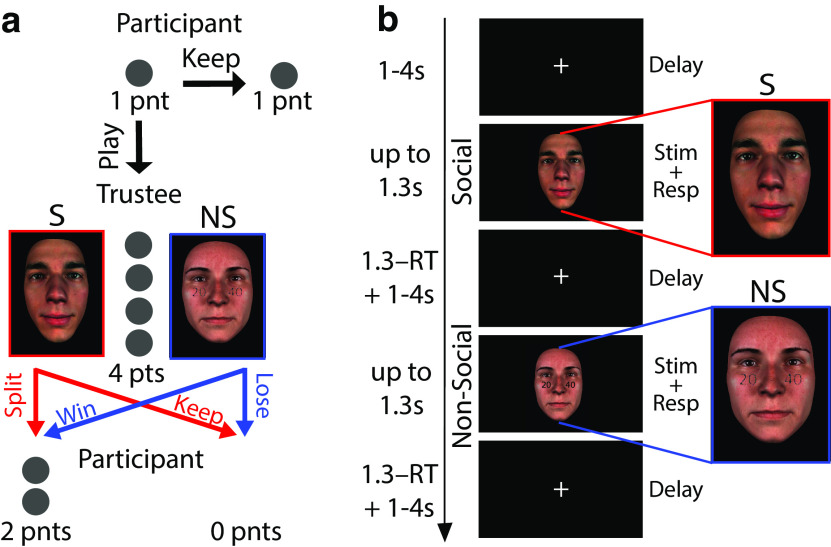
Experimental design. ***a***, A variant of the traditional Trust Game in which a participant (Investor) is allocated one point and they need to decide whether to “Keep” the point or “Play” for the chance of winning two points. During “Play” choices, the one point is quadrupled and passed on to a Trustee, which takes the form of either a social agent (S;red) or a purely probabilistic gamble (NS; blue). The Trustee can either split the four points evenly and give the participant two points or keep all four points to themselves (i.e., the participants receive 0 points). In the social context the probability of winning is based on the trustworthiness of the social agent displayed in the stimulus, while in the nonsocial context by the reward probability range displayed on top of a face, neutral for trustworthiness. ***b***, Social (S; red outline) and nonsocial (NS; blue outline) experimental design trials. Each trial began with a variable duration (1–4 s) fixation cross screen, which served as an intertrial interval. The fixation screen was followed by a stimulus screen which remained available for up to 1.3 s, during which participants indicated their choice (“Play” or “Keep”). The stimulus screen was replaced by a fixation cross following choice for the remainder of the 1.3 s.

The experimental design consisted of three parts: (1) an initial behavioral session comprising a rating task and a separate choice task, (2) an online rating task 1 d before the main EEG-fMRI experiment, and (3) a rating task and the main choice task during which participants underwent scanning. All tasks were framed in the context of an economic game. Specifically, we used a variant of the trust game in which participants engaged in a series of one-shot trust games involving a Trustee and an Investor. In each game, the Trustee is allocated one point per trial and has two options: (1) to obtain a small but certain reward by keeping the one point (“Keep” option) or (2) to invest the one point with the Investor for a bigger (two points) but uncertain reward (“Play” option). When the Trustee chooses the latter option the one point is quadrupled and it is now up to the Investor to determine whether to keep all four points for themselves or split them evenly between the two players 2 (Fig. 2*a*).

During the rating tasks, we told participants that the face identities belonged to individuals who have previously taken part in an economic game (i.e., a trust game like the one described above) in which they were assigned the role of Trustee. The goal of the participants in these indirect trustworthiness rating tasks was to assess the face identities' social attitudes by estimating the overall likelihood with which each Trustee split the augmented endowment (in the range 0–1, on a continuous scale). This framing ensured that our social stimuli varied along the same scale of reward probability as nonsocial gambles (see below). This was a critical feature of our design since embedding the rating in the context of a trust game allowed us to circumvent the arbitrary nature of explicit trustworthiness ratings (e.g., using Likert scales) and ensured a direct mapping between social and nonsocial choices. Furthermore, this indirect measure of perceived trustworthiness was previously shown to be more ecologically valid compared with explicit ratings (Uleman and Kressel, 2013) and further ensured that trustworthiness judgments became the product of an economic decision as in our main experimental paradigm. Importantly, when instructing participants, we purposely avoided explicit mentions of “trustworthiness” to sidestep the possibility of participants developing unusual strategies in the game because of social desirability biases.

Correspondingly, we told participants that during the main choice task they would assume the role of the Investor themselves and play with the same face identities they encountered during the ratings tasks (in social trials) or using purely probabilistic gambles (in nonsocial trials). On each trial participants had to choose between the “Keep” and “Play” option, however the outcome of the trial depended on the context (Fig. 2*a*). In the social context, we told participants that the probability of doubling their points was based on one of the Trustee's responses from when they previously played the game in our lab. In reality, we used the participant-specific reports on the likelihood of individual face identities splitting the augmented endowment to construct reward probability ranges that were comparable to those used in the nonsocial contexts (via explicit reward probability values). This design ultimately ensured that participants' decisions in the main choice task would be based on the same economic considerations, that is, the reward probability associated with a “Play” choice, across both contexts. To make our cover story more realistic for our participants, we took pictures of their own faces and told them that their face displays and responses would be used for similar experiments in the future. In reality, we would delete the pictures after each session.

In the main choice task, we included five different levels of reward probabilities (given a “Play” choice): 0–0.2, 0.2–0.4, 0.4–0.6, 0.6–0.8, and 0.8–1. Ultimately, these ranges correspond to three broad task difficulty levels: easier trials favoring either a “Keep” or “Play” choice (i.e., 0–0.2 and 0.8–1, respectively), medium difficulty trials in which the outcome uncertainty for “Play” choices begins to increase (i.e., 0.2–0.4, and 0.6–0.8), and difficult trials for the most ambiguous set of reward probabilities (i.e., 0.4–0.6). In the social context, we assigned each of the face identities into the five bins based on the participants ratings on the day of the EEG-fMRI. We nonetheless used their ratings across all three ratings sessions to identify face identities that received inconsistent ratings across sessions (more than two bins apart) and removed them from the experiment (on average, 10.807 face identities were removed). On average, there were 23, 34, 32, 36, and 14 face identities across the five levels of reward probability, respectively.

In the nonsocial context, the reward probability was presented explicitly through a probability range displayed on a face neutral for trustworthiness (Fig. 1). We embedded a background face in the nonsocial trials to equalize the perceptual load across contexts to enable direct comparisons between contexts (e.g., when searching for domain-specific representations). Furthermore, the inclusion of a face stimulus in both conditions ensures that any domain-specific differences across conditions (i.e., based on task-dependent social or nonsocial features) are not driven merely by differences in the bottom-up processing of the stimuli themselves. We additionally distorted the number presenting the reward probability ranges to equalize the early encoding of the perceptual stimuli across contexts (by ensuring that reaction times (RTs) and nondecision times were comparable across contexts, during initial piloting of the task). During these trials, we instructed participant to focus exclusively and base their choices on the numbers displayed on the stimuli.

The main choice task during the initial behavioral session was virtually identical to the one used for the EEG-fMRI experiments, with the main difference being that participants received feedback following each of their choice on a trial-by-trial basis. We included this initial choice task to identify participants who would understand the task and to offer participants a chance to familiarize themselves with the main paradigm. We provided immediate feedback relating to their choice to reinforce the associations between the stimuli and the outcome. During the EEG-fMRI experiments participants would be informed of their accumulated points only during the breaks (after every 50 trials). Furthermore, to motivate participants to engage with the task, we told them that in addition to their base rate payment (behavioral session: £6, EEG-fMRI session: £16) they would receive a variable bonus (up to £4) based on the overall points they collected during the experiment. We did not provide further details on how points translated into monetary rewards. On average, the participants who were included in the final analysis received £8.03 ± 0.31 during their initial behavioral session. In the EEG-fMRI session, we paid all participants £20.

The main choice task during the EEG-fMRI session comprised of a total of 500 trials, split evenly across the social and nonsocial contexts. All trials were presented in fully interleaved fashion and they were further broken down into five runs, lasting ∼7 min each. In each run we included a 30-s break at the middle and the end (i.e., after every 50 trials). During each trial, a jittered fixation cross (1–4 s, mean = 2 s; optimized for maximizing discriminability across contexts as previously described (Mumford et al., 2015) would be followed by the presentation of the face stimulus (either social or nonsocial). For both contexts, the stimulus remained on screen until the participant made a response or for a maximum of 1.3 s. If the participant's response was faster than 1.3 s, the fixation cross reappeared for the remaining time up to 1.3 s, to keep the run times consistent between participants (Fig. 2*b*). Participants used an MR-compatible response box (Cambridge Research Systems, 2019) to indicate their choices.

### Choice probabilities

To assess the similarity between the probabilities of “Play” choices across the social and nonsocial contexts, we used a conventional likelihood-ratio test. Specifically, we examined whether a single sigmoid curve (Weibull function) would fit the combined social and nonsocial choice data across the five reward probability levels better than two separate curves (Philiastides and Sajda, 2006). We performed this separately for each participant by fitting the best single Weibull function jointly to the two datasets in addition to the individual fits. The likelihoods (L) obtained from this procedure were transformed using the following equation:

(1) $$\lambda =-2ln\frac{\frac{1}{N}\sum_{i=1}^{N}L_{i}(\text{data }|\text{ joined curve})}{\frac{1}{N}\sum_{i=1}^{N}L_{i}(\text{data }|\text{ individual curves})},$$

where *N* represents the number of participants and λ is distributed as χ^2^ with 2 df (Hoel et al., 1971). If λ exceeds the criterion value (for *p* = 0.05), we concluded that a single function fits the data better than two separate domain-specific functions.

### Sequential sampling modeling

**Figure 3.**
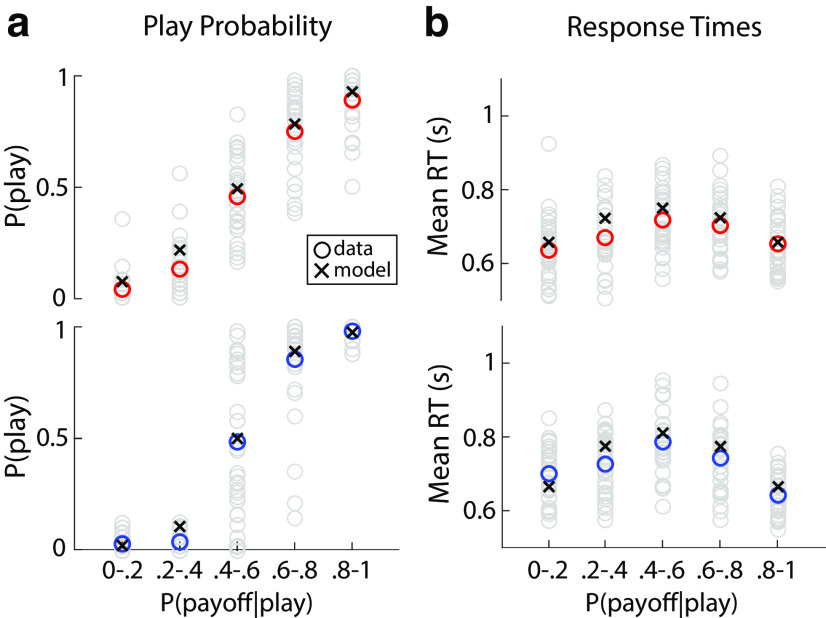
Social and nonsocial behavioral responses (red and blue circles) versus modeling performance of a drift diffusion model (black crosses) for proportion of “Play” choices (***a***) and response times (RTs; ***b***). “Play” responses increased with probability of reward given a “Play” choice [P(payoff—play)] and RTs were the highest when there was no strong evidence for or against “Play” decisions. Participant-specific behavior presented in gray circles.

We modeled the behavioral data using a special case of the leaky competing accumulator model (Fig. 3). Specifically, we used an Ornstein–Uhlenbeck process to model the evidence accumulation (EA) stage as previously described (Polanía et al., 2014; Pisauro et al., 2017):

(2) EA(t + 1)=EA(t) + (λEA(t) + k(evidence)dt + N(0,σ))+ bias(evidence=0).

We set the decision thresholds for “Play” and “Keep” choices to +1 and –1, respectively, such that positive drift rates are associated with reward probability levels favoring “Play” choices while negative drift rate values with those favoring “Keep” choices. Correspondingly, in Equation 2 the evidence represents a transformed version of the original five reward probability levels such that they are now centered around zero (i.e., [−0.5 −0.25 0 0.25 0.5]).

Free parameter *k* modulates the input, λ represents the acceleration to threshold and *N*(0, σ) is a Gaussian noise term with standard deviation σ. We used a time increment dt = 0.001s and assumed that the model makes a decision when |EA|> boundary. Early visual encoding and motor preparation were captured by the nondecision time free parameter (nDT), which was included into the total reaction time (RT). For the indecision point (i.e., 0 evidence) we included an extra free parameter, *bias*, to account for potential interindividual biases toward either “Play” or “Keep” choices. We separated the RTs according to the selected action (“Keep” or “Play”). We then combined the RTs from both trial types into a single distribution by flipping the sign of the “Keep” trials, so that all the times in this distribution received a negative sign (Voss et al., 2004). This RT distribution and participants' choice probabilities were compared with the RT distribution and proportion “Play” choices generated by the model (Fig. 3). For a given set of parameter estimates, we estimated the log likelihood (LL) of the data using the following formula:

(3) $$\begin{array}{l} LL∼\sum_{evidence=1}^{5}log(KS(RT_{data}^{evidence},RT_{model}^{evidence}))\\ +\sum_{evidence=1}^{5}log(exp(-{\left(\frac{Pplay_{data}^{evidence}-Pplay_{model}^{evidence}}{0.01}\right)}^{2})). \end{array}$$

*KS*(p,q) is used to estimate the probability that two distributions are equal, based on the Kolmogorov–Smirnov test (via the ktest2 function in MATLAB). *Pplay* represents the fraction of “Play” choices for each of the five levels of evidence. To fit the model, we used a two-step procedure. First, we used the fmincon MATLAB function to provide an initial estimate of participant-specific parameters. Specifically, we ran this procedure 20 times and the parameters associated with the smallest LL were selected for the next step. Secondly, we ran a grid search fitting procedure for each participant using a fine-grained parameter space around the estimates obtained in the previous step. Choices and RT distributions were created for each possible combination of the four free parameters from 5000 simulated decision traces for each context.

Mean parameter estimates for the social context: λ: 5.774 ± 2.357, k: 3.206 ± 1.555, σ: 0.02 ± 0.01, *bias*: −0.00,004 ± 0.0004, nDT: 0.336 ± 0.09. Mean parameter estimates for the nonsocial context: λ: 5.277 ± 2.37, k: 2.611 ± 1.355, σ: 0.011 ± 0.006, *bias*: −0.00,002 ± 0.0006, nDT; 0.304 ± 0.089. For the majority of parameters, there was no significant differences between the two contexts (λ: *t*_(30)_ = −1.3, two-sided *p* = 0.203, bias: *t*_(30)_ = 0.26, two-sided *p* = 0.8, k: *t*_(30)_ = −1.349, two-sided *p* = 0.188, nDT: *t*_(30)_ = −1.363, two-sided *p* = 0.183), reinforcing the notion that choice behavior was comparable across social and nonsocial choices. There was a significant difference in the noise term (σ: *t*_(30)_ = −4.244, two-sided *p* < 0.001), likely because of additional internal variability in processing the faces and their trustworthiness in the social context compared with processing the numbers in the nonsocial trials. We expect that these differences would be captured in the trial-by-trial variability reflected in our EEG slopes, which we used to inform the fMRI analysis and identify the brain nodes correlating with the EA process (see below).

Finally, although we did not include an explicit urgency manipulation in our design, we also tested a variant of our model with a variable boundary parameter across the two contexts. We performed a formal model comparison with the original model by calculating subject-specific BIC scores and found that the addition of an extra free parameter led to higher summed BIC scores than the original model and thus, worse fits (Summed nonsocial BICs in fixed boundary model: 5896.265, and in the variable boundary model: 6180.934; Summed Social BICs in the fixed boundary model: 3519.885, in the variable boundary model: 3568.106). This further ensures that the original drift rate estimates, which we use to establish a link to the process of EA in the EEG, are not reflective of any unaccounted variance in other decision-related parameters. We further note that since our fMRI analysis is based purely on EEG-derived slopes of EA (see below), we are effectively circumventing any remaining issues with model estimation/mis-specification.

### EEG data acquisition

We used an MR-compatible EEG amplifier system (Brain Products) to collect the data and Brain Vision Recorder software (Brain Products) to continuously record EEG at 5000 Hz. A hardware 0.016- to 250-Hz bandpass filtered the data online. We placed the 64 Ag/AgCl scalp electrodes according to the 10–20 system, with the reference and ground electrodes being built in between electrodes Fpz and Fz and between electrodes Pz and Oz, respectively. Each electrode had in-line 10-kΩ surface-mount resistors to ensure subject safety, which was further guaranteed by bundling and twisting all leads for their entire length. We lowered the input impedance for each electrode to <50 kΩ (25 KΩ average across participants). The acquisition of EEG and MRI data were synchronized (Syncbox, Brain Products) and MR-scanner triggers were recorded separately for the subsequent offline removal of MR gradient artifacts. To facilitate the recording of the scanner triggers, the scanner pulses were lengthened to 50 μs via an in-house pulse stretcher. Experimental event codes and participants' responses were synchronized, and recorded simultaneously, with the EEG data through the Brain Vision Recorder software. We positioned subjects inside the scanner by ensuring that electrodes Fp1 and Fp2 were aligned with the isocenter of the MR scanner. Finally, the cabling connecting to the EEG amplifiers at the back of the bore was secured to a cantilever beam to minimize scanner vibration artifacts.

### EEG data preprocessing

We used MATLAB (MathWorks) to preprocess and analyze the EEG data. EEG signals recorded inside an MR scanner are contaminated primarily with MR gradient artifacts and ballistocardiogram (BCG) artifacts because of magnetic induction on the EEG leads. To correct for gradient-related artifacts, we constructed average artifact templates from sets of 70 consecutive functional volumes centered on each volume of interest, and subtracted these from the EEG signal. This process was repeated for each functional volume in our dataset. To remove any residual spike artifacts we applied a 12-ms median filter. Furthermore, we applied a 0.5- to 20-Hz bandpass filter to remove slow DC drifts and higher frequency noise. All data were downsampled to 1000 Hz.

To remove eye blinks, we asked participants to perform an eye movement calibration task before the main experiment during which they were instructed to blink repeatedly several times while a central fixation cross was displayed in the center of the computer screen. We recorded the timing of these events and used principal component analysis to identify linear components associated with eye-blinks, which were subsequently removed from the broadband EEG data collected during the main task (Parra et al., 2005).

BCG artifacts share frequency content with the EEG and are therefore more challenging to remove. To avoid loss of signal power in the EEG we only removed a small number of participant-specific BCG components using principal component analysis and relied instead on our multivariate discriminant analysis (see single-trial EEG analysis section below) to identify task-related discriminating components that are likely to be orthogonal to the BCG (Fouragnan et al., 2015; Gherman and Philiastides, 2018). This approach is robust to the presence of BCG artifact residuals, specifically, because of the (spatial) multivariate nature of our classification techniques.

Correspondingly, we first extracted BCG principal components from the data after low-pass filtering at 4 Hz (i.e., to extract the signal within the frequency range where BCG artifacts are typically observed) and then created datasets with different number of principal components removed (up to 5). The sensor weightings corresponding to the relevant components were projected onto the broadband data and subtracted out. We determined the number of optimal principal components for each participant by identifying peak classification performance along the task-relevant dimension (see below) using cross validation (average number of BCG components across participants: 2.447 ± 1.969).

### Single-trial EEG analysis

As we aimed to examine whether social and nonsocial choices share a common underlying mechanism for integrating relevant decision evidence, we leveraged the high temporal resolution of the EEG data to identify signals exhibiting a gradual build-up of activity consistent with a general process of EA (Polanía et al., 2014; Pisauro et al., 2017). We hypothesize that if such signals exist, we should observe reliable ramp-like activity with a build-up rate that is proportional to the amount of decision difficulty.

**Figure 4.**
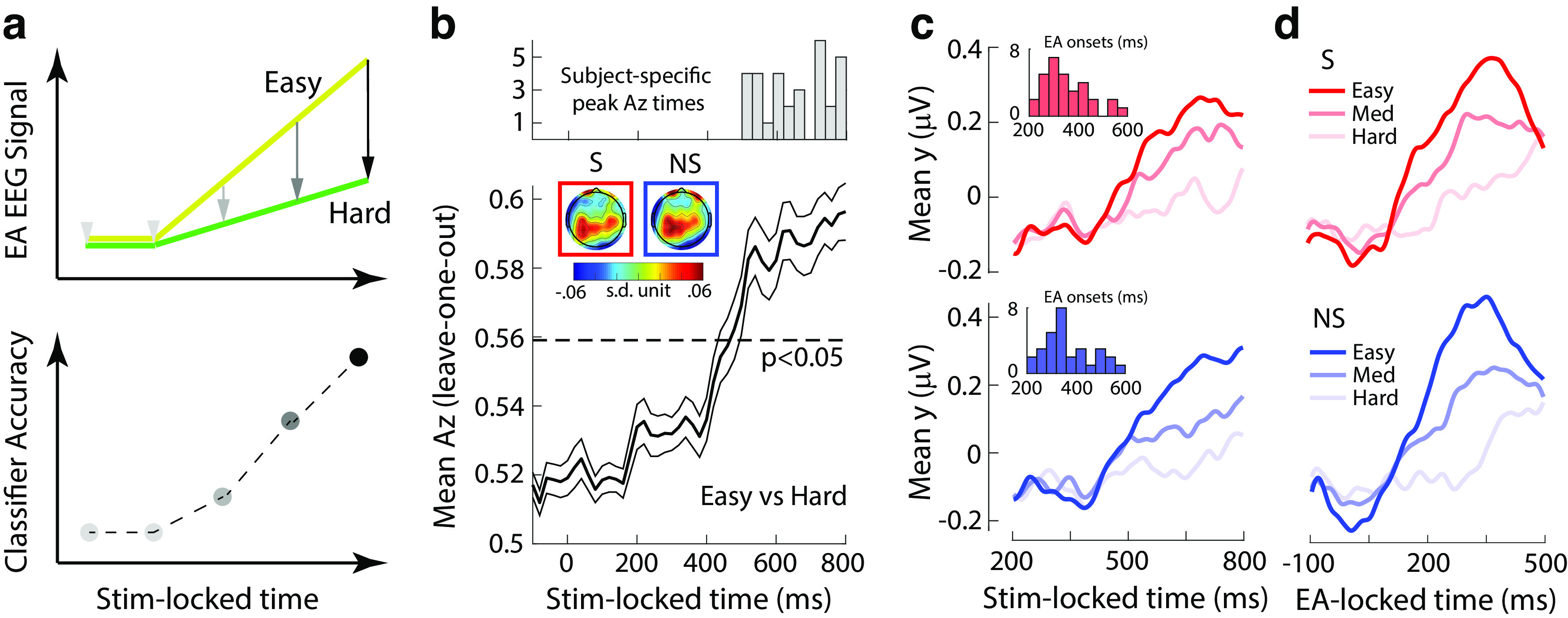
Linear discriminant analysis of the EEG. ***a***, Build-up rates for hypothetical evidence accumulation (EA) signals for easy (yellow) and difficult (green) trials (top) and how differences in the rate of EA could manifest in the accuracy of an EEG classifier trained on stimulus-locked data. ***b***, Average discrimination performance (Az; using leave-one-out cross validation) between easy and difficult trials across participants along with histogram of participant-specific peak discrimination times (top). The dashed line represents the average Az value leading to a significance level of *p* = 0.05, estimated using a separate bootstrap test. The thinner black lines indicate standard errors of the mean across participants. Insets, Scalp topographies (forward models) of the discriminating activity estimated at time of peak discrimination averaged across participants for the social (S; red outline) and nonsocial (NS; blue outline) trials. ***c***, The average temporal profile of the discriminating activity across participants (obtained by applying the participant-specific classification weights estimated at a time of peak discrimination) for the three levels of decision difficulty for social (red) and nonsocial trials (blue), locked to the onset of the stimulus onset. Insets, Histograms of participant-specific EA onset times for social (red) and nonsocial trials (blue). ***d***, The average temporal profile of the discriminating activity across participants, realigned to the onset of EA as estimated in **c**, for the three levels of decision difficulty for social (red) and nonsocial trials (blue).

To identify activity related to EA we used a single-trial multivariate linear discriminant analysis (LDA; Parra et al., 2005; Sajda et al., 2009) to discriminate between easy (i.e., reward probabilities 0–0.2 and 0.8–1) and difficult trials (reward probabilities 0.4–0.6) in stimulus-locked EEG data, collapsing across both social and nonsocial trials. Such a persistent accumulating activity with a build-up rate proportional to the amount of decision difficulty should lead to a gradual increase in the classifier's performance while the traces for the easy and difficult trials diverge as a function of elapsed time in stimulus-locked data (Fig. 4*a*). We treated the medium difficulty trials (i.e., reward probabilities 0.2–0.4 and 0.6–0.8) as “unseen” data, to more convincingly test for a full parametric effect on the build-up rate associated with the different levels of decision difficulty (see below).

More specifically, our method estimates an optimal combination of EEG sensor linear weights (i.e., a spatial filter **w**) which, applied to the multichannel EEG data [*x*(*t*)], yields a one-dimensional projection [i.e., a discriminant component *y*(*t*)] that discriminates between the two contexts:

(4) $$y(t)=w^{T}x(t)=\sum_{i=1}^{D}w_{i}x_{i}(t),$$

where *D* represents the number of channels, indexed by *i*, and *T* indicates the transpose of the matrix. We applied this method to identify **w** for short (60 ms) overlapping time windows centered at 20-ms interval time points, between −100 and 800 ms relative to the onset of the decision stimulus. This procedure was repeated for each subject and time window separately. By integrating information spatially across the multidimensional sensor space, we increase signal-to-noise ratio while simultaneously preserving the trial-by-trial variability in the relevant discriminating component. More specifically, applied to an individual trial, spatial filters (**w**s) obtained in this way produce a measurement of the discriminant component amplitude for that trial, which we treat as a neural surrogate of the relevant decision variable that is being integrated.

To estimate the optimal discriminating spatial weighting vector **w** we used a regularized Fisher discriminant analysis as follows: $w=S_{c}(m_{2}-m_{1})$, where *m_i_* is the estimated mean of condition i and $S_{c}=1/2(S_{1}+S_{2})$ is the estimated common covariance matrix (i.e., the average of the condition-wise empirical covariance matrices, $S_{i}=1/(n-1)\sum_{j=1}^{n}\left(x_{j}-m_{i}\right){\left(x_{j}-m_{i}\right)}^{T}$, with *n* = number of trials). We replaced the condition-wise covariance matrices with regularized versions of these matrices to counteract potential estimation errors: ${\tilde{S}}_{i}=(1-\lambda )S_{i}+\lambda \nu I$, with λ ∈ [0, 1] being the regularization term and ν the average eigenvalue of the original *S_i_* [i.e., *trace*(*S_i_*) /*D*, with *D* corresponding to the dimensionality of our EEG space]. Note that λ = 0 yields unregularized estimation and λ = 1 assumes spherical covariance matrices. Here, we optimized λ for each participant using leave-one-trial-out cross validation with the following λ values ∈ [0, 0.01, 0.02, 0.04, 0.08, 0.16] (λ mean ± SE: 0.067 ± 0.072).

To quantify the performance of the discriminator for each time window, we computed the area under a receiver operating characteristic (ROC) curve (i.e., the *A_z_* value), using a leave-one-trial-out cross-validation procedure. Specifically, for every iteration, we used N–1 trials to estimate a spatial filter (**w**), which we then applied to the remaining trials to obtain out-of-sample discriminant component amplitudes [*y*(*t*)]. We used these out-of-sample amplitudes to compute the *A_z_*. In addition, we determined participant-specific *A_z_* significance thresholds (rather than assuming an *A_z_* = 0.5 as chance performance) using a subsequent bootstrap analysis whereby trial labels were randomized and submitted to the leave-one-trial-out test described above. This randomization procedure was repeated 500 times, producing a probability distribution for *A_z_*, which we used as reference to estimate the *A_z_* value leading to a significance level of *p* < 0.05 (Fig. 4*b*).

To produce the full temporal profile of the relevant discriminating components [*y*(*t*)], we applied the spatial filter **w** of the window associated with the highest discrimination performance (i.e., subjected the data through the “spatial generators” leading to the most reliable discrimination) across the entire stimulus-locked window (−100 to 800 ms poststimulus) and separately for each of the social and nonsocial contexts as well as the three difficulty conditions (easy, medium, and difficult; Fig. 4*c*). The times course of these discriminating components were then *Z*-scored separately for each participant and for each of the social and nonsocial contexts.

This procedure allowed us to investigate the gradual build-up of EA activity leading up to a point of peak discrimination and to extract the corresponding single-trial build-up rates used in subsequent analyses. These build-up rates (or slopes) were computed through a linear regression between onset and peak time of the accumulating activity extracted on a participant-specific basis. Specifically, we identified the time point at which the discriminating activity began to rise monotonically after an initial dip in the stimulus-locked data following any early evoked responses present in the data (onset time mean ± SE: 363.097 ± 97.046 ms for social trials and 376.161 ± 107.155 ms for nonsocial trials).

Finally, the linearity of our model also allowed us to compute scalp topographies of the relevant discriminating components from Equation 4 by estimating a forward model:

(5) $$a=\frac{Xy}{y^{T}y}.$$

The EEG data **X** and discriminating components **y** are now depicted in matrix and vector notation, respectively, for convenience. Equation 5 represents the electrical coupling of the discriminating component **y** that explains most of the activity in **X**. Specifically, strong coupling is linked to low attenuation of the component and can be visualized as the intensity of vector **a**. We estimated forward models for the resulting discriminating activity separately for social and nonsocial trials (Fig. 4*b*).

### Single-trial regression analyses

To examine the association between the probability of reward (i.e., indirect trustworthiness levels and pure probabilities in the social and nonsocial contexts, respectively) and the probability of playing (1: “Play”, 0: “Keep”) on individual trials (Fig. 3*a*) we performed the following single-trial logistic regression analysis (separately for each participant and for each of the social and nonsocial trials):

(6) $$P_{play}={\left[1+e^{-(\beta_{0}+\beta_{1}\times y(\text{reward probability}))}\right]}^{-1}$$

To examine the association between task difficulty [i.e., 1: easy (reward probabilities 0–0.2 and 0.8–1), 2: medium (reward probabilities 0.2–0.4 and 0.6–0.8), 3: difficult (reward probabilities 0.4–0.6)] and response times (RT) on individual trials (Fig. 3*b*), we performed the following single-trial regression analysis (separately for each participant and for each of the social and nonsocial trials):

(7) $$RT=\beta_{0}+\beta_{1}\times (\text{difficulty level}).$$

**Figure 5.**
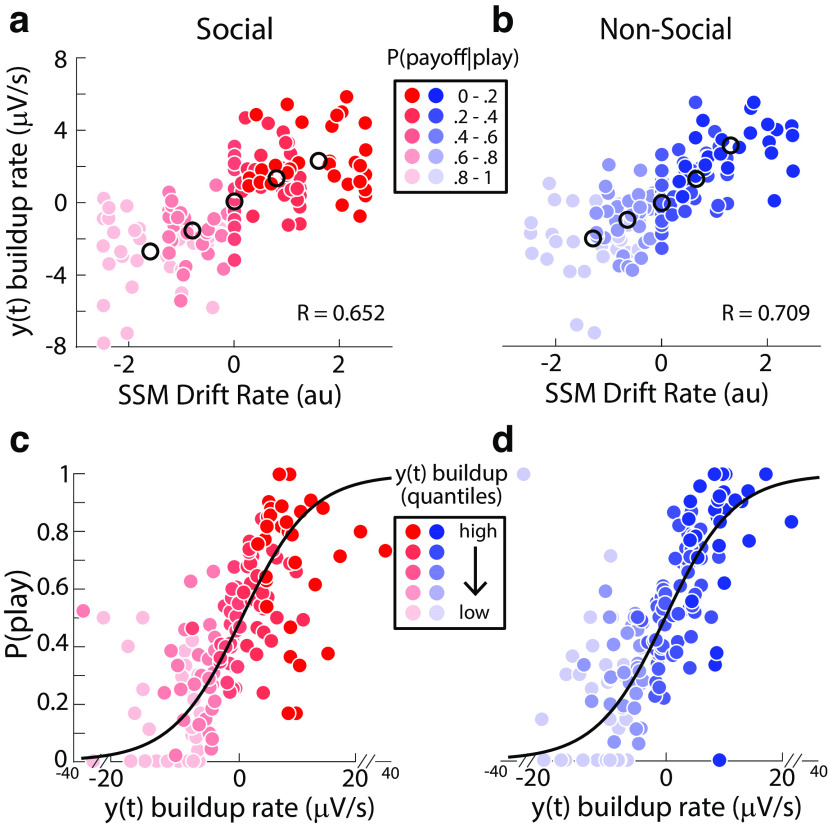
Linking EEG signatures of evidence accumulation to behavior. ***a***, ***b***, Participant-specific EA slopes [y(t) build-up rate] for each of the five levels of P(payoff|play) scale positively with SSM estimates of drift rate for both the social (***a***) and nonsocial (***b***) contexts. The EEG-derived EA signal y(t) from which the EA slopes were derived was normalized per trial to factor out any effects unrelated to the EA processing, such as attentional drifts. Black circles indicate population averages. ***c***, ***d***, Trial-by-trial estimates of EA slopes correlate positively with the probability of playing (Eq. 3) for both the social (***c***) and nonsocial (***d***) contexts. To visualize this association the data points were computed by grouping trials into five bins based on the EA slope estimates. Importantly, the black curves are derived from fits of Equation 3 to individual trials.

To offer further validation that the EEG signals we identified through our LDA analysis (independently from the behavioral model estimation) were related to the process of EA, we tested the extent to which the build-up rate of the EEG EA signals [i.e., *y*(*t*)] would correlate with sequential sampling model estimates of drift rate that were derived purely from participants' behavior (i.e., on fraction of “Play” choices and RTs; Fig. 3). To this end, we flipped the sign of the EEG slopes in the two reward probability levels which support “Keep” choices [i.e., P(payoff|play)={0−0.2,0.2−0.4}], since the “Play” and “Keep” choices were mapped to +1 and –1, respectively (see section on Sequential sampling model for details), and therefore positive (negative) drift rates reflected reward probability levels favoring a “Play” (“Keep”) choice (Fig. 5*a*,*b*).

To further examine the association between the rate of EA derived from the neural data and single-trial behavioral performance on the task (rather than mean drift rates from the model as in the analysis above), we ran a single-trial logistic regression analysis. Specifically, we used the trial-wise estimates of the slope of the EEG-derived EA signal to predict the probability of playing (1: “Play”, 0: “Keep”) on individual trials (Fig. 5*c*,*d*). Consistent with the previous analysis we flipped the sign of the EEG slopes in the two reward probability levels which support “Keep” choices. We expected, high positive and high negative EA rates to reflect easy “Play” and “Keep” choices, respectively, with intermediate magnitude slopes reflecting medium difficulty choices and slopes near zero representing difficult choices. We performed this analysis separately for each participant and for each of the social and nonsocial trials:

(8) $$P_{play}={\left[1+e^{-(\beta_{0}+\beta_{1}\times y(\text{buildup rate}))}\right]}^{-1}$$

Finally, we also ran a logistic regression predicting the probability of a “Play” choice using a combination of the EA-derived slopes and the probability of reward (as described above) to examine whether our neural estimates offer additional explanatory power, beyond what could be inferred by accounting for the overall experimental manipulation of task difficulty:

(9) $$P_{play}={\left[1+e^{-(\beta_{0}+\beta_{1}\times y(\text{buildup rate})+\beta_{2}\times y(\text{reward probability}))}\right]}^{-1}$$

In all four regression analyses, we tested whether the regression coefficients across participants (β_1_ values in Eqs. 6–8) came from a distribution with a mean different from zero (using separate two-tailed *t* test). We also compared the deviance scores associated with the three analyses predicting the choice behavior to assess whether the addition of the neural predictor would explain more of the variability in the data.

### MRI data acquisition

A Siemens 3-Tesla TIM Trio MRI scanner (Siemens) with a 12-channel head coil was employed for the (f)MRI acquisition. A T2*-weighted gradient echo was used to acquire functional volumes with an echo-planar imaging sequence (32 interleaved slices, gap: 0.3 mm, voxel size: 3 × 3 × 3 mm, matrix size: 70 × 70, FOV: 210 mm, TE: 30 ms, TR: 2000 ms, flip angle: 80°). We recorded five experimental runs of 205 whole-brain volumes each. Afterwards, we acquired phase and magnitude field maps (3 × 3 × 3 mm voxels, 32 axial slices, TR = 488 ms, short TE = 4.92 ms, long TE = 7.38 ms) for distortion correction of the acquired EPI images. Finally, a high-resolution anatomic volume was taken using a T1-weighted sequence (192 slices, gap: 0.5 mm, voxel size: 1 × 1 × 1 mm, matrix size: 256 × 256, FOV: 256 mm, TE: 2300 ms, TR: 2.96 ms, flip angle: 9°), which was used as an anatomic reference for the functional scans.

### fMRI data preprocessing

To guarantee a steady-state fMRI we removed the first five volumes per run and we used only the remaining 200 volumes for the analysis. Head-related motion correction, slice-timing correction, high-pass filtering (>100 s), and spatial smoothing (with a Gaussian kernel of 5-mm full-width at half maximum) were performed using the FMRIB's Software Library (Functional MRI of the Brain). The motion correction preprocessing step generated motion parameters which were subsequently included as regressors of no interest in the general linear model (GLM) analysis (see fMRI analysis below). Brain extraction of the structural and functional images was performed using the Brain Extraction tool (BET). The echo-planar imaging data for each participant was transformed into the subject-specific high-resolution space using a boundary-based registration (BBR) algorithm. The images were then registered to standard space (Montreal Neurologic Institute, MNI) using FMRIB's Nonlinear Image Registration tool with a resolution warp of 10 mm and 12 df. Finally, to correct for signal loss and geometric distortions because of B0 field inhomogeneities B0 unwarping was used for 29 out of 31 participants. Field map images were not acquired for the remaining two participants.

### fMRI analysis

**Figure 6.**
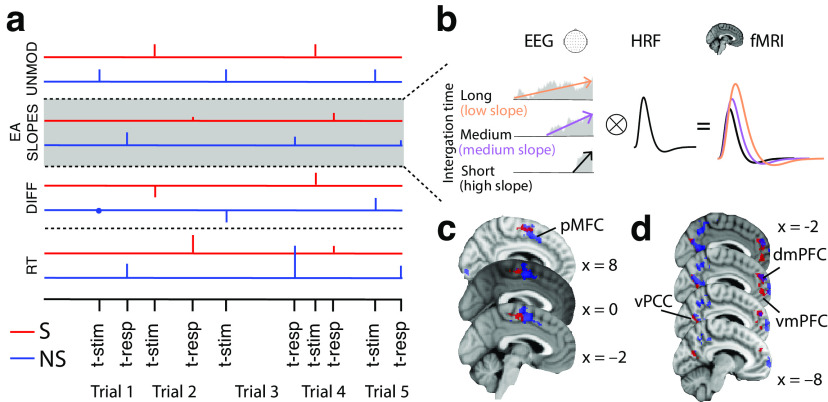
EEG-informed fMRI analysis. ***a***, The fMRI GLM model included two parametric boxcar regressors capturing the electrophysiological trial-by-trial variability in the slope of EA for each of the social and nonsocial trials at the time of decision. To absorb the variance associated with other task-related processes, for each of the social and nonsocial trials separately, we included the following additional regressors: UNMOD, two unmodulated (amplitude 1) boxcar regressors at the onset of the stimuli; DIFF, two parametric boxcar regressors of task difficulty (−1: hard, 0: mid, 1: easy); and RT, two parametric boxcar regressors modulated by individual trial RTs at the time of decision. All regressors had a fixed duration of 100 ms. ***b***, Example EA traces with different build up rates (colored arrows). Convolving these traces with a hemodynamic response function (HRF) leads to higher predicted fMRI activity for longer compared with shorter integration times, that is, higher predicted fMRI activity for shallower compared with steeper EA slopes. ***c***, According to the hypothesized prediction in ***b*** the EEG-informed fMRI predictors of the slope of EA revealed an activation in posterior-medial frontal cortex (pMFC) for both social and nonsocial trials. ***d***, We found that the pMFC showed task-dependent co-activation with regions of the human valuation system, specifically clusters along the medial wall of the prefrontal cortex as well as regions of the posterior cingulate cortex. The clusters represent mixed-effects activations that survived |Z|>2.57 and that were cluster-corrected (*p* < 0.05) using a resampling procedure with a minimum cluster size of 88 voxels (see Materials and Methods). vmPFC: ventromedial profrontal cortex; dmPFC: dorsomedial prefrontal cortex; vPCC: ventral posterior cingualte cortext.

Here, we aimed to exploit endogenous trial-by-trial variability in the slope of EA to create EEG-informed fMRI predictors to identify candidate regions for the process of EA in social and nonsocial contexts (Fig. 6*a*). More specifically, we used the trial-wise changes in the rate of EA, which embody the momentary changes in the decision process as it unfolds, to predict BOLD activity in the fMRI signal. Note that, unlike conventional event-related potentials for which the trial-by-trial signal fluctuations might be contaminated from unspecific neural process, the nature of our multivariate EEG discriminant analysis ensured these processes were effectively subtracted out, facilitating instead the spatial “unmixing” of the main signal of interest (i.e., process of EA; Ratcliff et al., 2009; Philiastides et al., 2014).

We performed whole-brain statistical analyses of the functional data using a multilevel approach within the framework of a GLM, as implemented in FSL (using the FEAT module; S.M. Smith et al., 2004):

(10) $$Y=x\beta +\epsilon =\beta_{1}X_{1}+\beta_{2}X_{2}+...+\beta_{N}X_{N}+\epsilon$$

Y represents the time series (with T time samples) for a voxel and X is a T × *N* design matrix where the columns correspond to the different regressors included in the design (see below) convolved with a canonical hemodynamic response function (double-γ function). β is a *N* × 1 column vector of regression coefficients and ϵ a T × 1 column vector of residual error terms. We performed a first-level analysis to analyze each participant's individual runs, which we then combined using a second-level analysis (fixed effects). We combined data across participants using a third-level, mixed-effects model (FLAME 1), treating participants as a random effect. Time-series statistical analysis was conducted using FMRIB's improved linear model with local autocorrelation (Woolrich et al., 2005).

Our GLM included four regressors of interest for each of the social and nonsocial contexts (i.e., a total of eight regressors). More specifically, for each of the social and nonsocial trials, we included (1) an EEG-informed regressor with a parametric amplitude modulation based on the trial-by-trial fluctuations in the rate of EA [i.e., trial-wise slopes in *y*(*t*)]; (2) a parametric regressor with amplitude modulation based on individual trial RTs; (3) a parametric regressor with amplitude modulation based on the individual trial task difficulty, not accounted for by the EEG-derived regressor (−1: difficult, 0: medium, 1: easy); and (4) an unmodulated regressor (i.e., all amplitudes set to 1) to account for any additional unaccounted variance in the data, which specifically aimed to capture activity in early evidence representations independent of any other task-related manipulations (Fig. 6*a*).

We note that the EA slopes derived from the EEG were not highly correlated with individual RTs (Social: *r* = −0.297, Nonsocial: *r* = −0.333). This is because of the high degree of intertrial variability in the decision and motor planning stages, as has been demonstrated consistently in previous modeling and experimental studies (Ratcliff et al., 2009; Philiastides et al., 2014; Verdonck et al., 2021). As such, RTs do not constitute a major confounding factor, but we nonetheless included separate nuisance RT predictors in our fMRI analysis. We modeled all regressor events as boxcar functions (i.e., duration 100 ms). For the first two regressors, event onset times were aligned to the time of response, while for the last two, to the onset of stimulus presentation. Using the unmodulated regressors we also computed standard contrast and conjunction maps between social and nonsocial trials. Finally, we added the motion correction parameters obtained from fMRI preprocessing (three rotations and three translations) as additional covariates of no interest.

Our EEG-informed fMRI approach benefits from using the actual neural signals, which could capture latent variability in information processing that might otherwise go amiss when using simple behavioral or model-derived indices (Sajda et al., 2011; Philiastides et al., 2021). For example, most sequential sampling models only produce mean estimates of the relevant decision variables (e.g., drift rate) across many trials, with only few studies attempting to derive single-trial parameter estimates (Turner et al., 2015; Gluth et al., 2017). Here, instead, we estimate the rate of EA on individual trials purely from the slope of the accumulating activity we identified in the EEG data. This way we could account for true endogenous variability in EA, that might not be reflective in behavior (and hence the model) and circumvent potential issues related to model estimation and/or mis-specification when deriving build-up rates purely based on fits to behavior.

### Resampling procedure for fMRI thresholding

In order to establish a reliable significance threshold for the fMRI data, while properly correcting for multiple comparisons, we used a resampling procedure, which examines a priori statistics of the trial-wise variability in the parametrically adjusted regressors (i.e., regressors 1–3 above) in a way that trades off cluster size and maximum voxel *Z*-score (Fouragnan et al., 2015; Gherman and Philiastides, 2018). Specifically, for each resampled iteration, we maintained the onset and duration of the regressors identical, while shuffling the amplitude values across trials, runs and participants. Thus, the resulting regressors for each participant were different as they were constructed from a random sequence of regressor amplitude events. This procedure was repeated 100 times and for each iteration we performed the full three-level analysis (run, participant, and group). Finally, we estimated a joint threshold for the cluster size and *Z*-score based on the cluster outputs per shuffled regressor. This was achieved by constructing a null distribution for this joint threshold based on the size of all clusters larger than 10 voxels and with *Z*-scores larger than —2.57— (i.e., considering both positive and negative correlations) across all shuffled regressors. We found that the largest 5% of cluster sizes exceeded 88 voxels. We therefore used these results to derive a corrected threshold for our statistical maps, which we then applied to the clusters observed in the original data (that is, Z = ±2.57, minimum cluster size of 88 voxels, corrected at *p* = 0.05).

### Psychophysiological interaction (PPI) analysis

We conducted a psychophysiological interaction (PPI) analysis to probe the functional connectivity between the pMFC, which was found to correlate with the trial-by-trial variability in our EEG-informed EA regressor, and the rest of the brain. To carry out the PPI analysis, we first extracted time-series data from group-level activation clusters in the pMFC (seed), separately for each of the social and nonsocial contexts. Specifically, we identified the relevant pMFC clusters that were situated within the supplementary motor area (SMA) portion of the cluster and were most consistent with previous reports of EA-related activity in this region (Pisauro et al., 2017) and then back-projected these clusters from the group (standard) space into the individual participant's EPI (functional) space (by applying the inverse transformations estimated during the main registration procedure). The average time-series data from the back-projected voxels, which displayed activations in the direction of the predicted EA profile were then used as the physiological regressor in our PPI analysis.

The main aim of this analysis was to investigate potential task-dependent associations between the site of EA and regions involved in domain-general value computations. If such an association exists, the coupling between these regions should be stronger while the process of EA unfolds and it should also scale with the difficulty of the decision. To this end, our psychological regressor was constructed as a parametric boxcar regressor, the amplitude of which reflected the difficulty (1 = difficult, 2 = medium, 3 = easy) and the duration of which reflected the RT of each trial. We expected the relevant coupling to be negative, as easier trials decrease integration times and correspondingly the overall integrated activity (that is, area under the accumulation curve; Fig. 6*b*). The resulting fMRI statistical maps were corrected based on the threshold derived from the resampling procedure described above.

### Data and code availability

The data and code required to reproduce the main findings are available at https://osf.io/hrgp5/?view_only=c84a4a66aebd4284951c8efd3a4d65fd.

## Results

We investigated economic decisions within a social context by exploiting trustworthiness in a partner's face during a strategic economic game to generate predictions about possible outcomes and within a nonsocial (purely probabilistic) context by manipulating outcome probabilities in individual gambles. Importantly, we created parametrically modulated stimuli along comparable scales of reward probability in each of the social and nonsocial contexts (while keeping reward magnitude constant across both contexts). The nonsocial stimuli were associated with a range of pure reward probabilities chosen from the full probability range (from 0 to 1), placed on top of a face image (neutral for trustworthiness) to equalize perceptual load across the social and nonsocial stimuli as well as to eliminate any potential confounds associated with differences in the bottom-up processing of the stimuli across contexts (Fig. 1; see Materials and Methods for more details).

We derived comparable reward probabilities for the social stimuli by asking participants (*N* = 31) to provide indirect trustworthiness ratings for a series of 150 face identities. Specifically, we framed this rating stage in the context of a trust game (Berg et al., 1995). Usually a trust game involves an interaction between two players, the Investor and the Trustee. The Investor decides whether to send a monetary endowment to the Trustee that gets multiplied by a certain factor (“Play” option) or to retain possession of the initial endowment (“Keep” option). In turn, the Trustee can decide whether or not to send a fixed share of the augmented amount back to the Investor so that both parties can benefit from the interaction. We told participants that each face belonged to people who had previously taken part in a similar study in the role of the Trustee, and we asked them to indicate the overall likelihood (in the range 0–1) that each person had returned a fixed share (50%) of the augmented endowment entrusted to them (Fig. 2*a*).

During the main (EEG-fMRI) task, participants assumed the role of the Investor in a series of one-shot trust games. In each game they had to decide whether to choose between a small but sure reward (one point; “Keep” option) or a bigger, but riskier payoff (two points; “Play” option). We randomly interleaved nonsocial trials (i.e., probabilistic gambles) in which we controlled the likelihood of obtaining the higher payoff with explicit reward probabilities, matched against subject-specific indirect trustworthiness ratings as highlighted above (Fig. 2*b*).

We instructed participants that the probability of receiving the higher payoff for “Play” choices would be based on the overall likelihood with which each face identify split the augmented endowment (here four points) in a previous study (social trials) or the pure reward probabilities depicted on the face stimuli (nonsocial trials). We sampled the full range of reward probabilities given a “Play” choice using five levels [P(payoff|play)={0−0.2,0.2−0.4,0.4−0.6,0.6−0.8,0.8−1}]. In social trials, we populated each reward probability level with face identities based on the subject-specific perceived trustworthiness from the initial rating stage.

### Behavioral performance in social and nonsocial contexts

Participants' fraction of “Play” choices (Eq. 6) correlated positively with the overall reward probability across both the social and nonsocial trials (Social: *t*_(30)_ = 17.769, two-sided *p* < 0.001; Nonsocial: *t*_(30)_ = 4.086, two-sided *p* < 0.001), indicating that they selected the riskier option more frequently as the likelihood of receiving the higher payoff increased (Fig. 3*a*). More importantly, we demonstrated that choice behavior was comparable across the social and nonsocial trials. Specifically, we used a likelihood-ratio test (see Materials and Methods) to show that a single sigmoid function (Eq. 1) fit the fraction of “Play” choices (jointly across both conditions) as well as two separate functions [λ(30) = 0.551, *p* = 0.759].

The mean response times (RTs) as a function of the overall reward probability exhibited an inverted V relationship, across both the social and nonsocial trials (Fig. 3*b*), consistent with a positive relationship with task difficulty (Social: *t*_(30)_ = 10.024, *p* < 0.001; Nonsocial: *t*_(30)_ = 10.692, *p* < 0.001). In other words, we observed the longest RTs for the most difficult trials (reward probabilities 0.4–0.6), the shortest RTs for the easiest trials (reward probabilities 0–0.2 and 0.8–1) and intermediate RTs for medium difficulty trials (reward probabilities 0.4–0.6 and 0.6–0.8). The overall RTs showed a small (41.637 ms), albeit significant difference between the social and nonsocial trials (paired *t* test: *t*_(30)_ = −3.274, two-sided *p* = 0.003), with social trials (*M_S_* = 677.864 ms, *SD_S_* = 86.479 ms) being on average faster than nonsocial ones (*M_NS_* = 719.502 ms, *SD_NS_* = 91.287 ms).

### Evidence accumulation in social and nonsocial contexts

Having established comparable behavioral performance across the social and nonsocial contexts, we asked whether these share a common underlying mechanism for integrating relevant decision evidence. To this end, we aimed to identify correlates of a general process of EA in our EEG data by looking for a reliable ramp-like activity with a build-up rate that is proportional to the amount of decision difficulty (Polanía et al., 2014; Pisauro et al., 2017).

We used a single-trial multivariate linear classifier (Parra et al., 2005; Sajda et al., 2009) designed to estimate spatial weightings of the EEG sensors that discriminate between easy versus difficult trials (see Materials and Methods). As hypothesized, the classifier's performance increased systematically over time, reflecting the potential divergence in the gradual build-up of activity between easy and difficult trials (Fig. 4*b*). On average, the classifier's performance began increasing after 400 ms poststimulus (i.e., after early encoding of the relevant evidence) and peaked several hundred milliseconds later.

The spatial distribution of this discriminating activity (i.e., forward model; see Materials and Methods) from participant-specific windows of peak discrimination between easy and difficult trials (Fig. 4*b*, top) revealed comparable centroparietal topographies across social and nonsocial contexts (*r* = 0.896, *p* < 0.01; Fig. 4*b*, inset). These similarities are suggestive of common neural generators across the two contexts, consistent with those reported previously in the perceptual domain (Kelly and O'Connell, 2013; Philiastides et al., 2014; Herding et al., 2019).

To formally characterize the temporal profile of the discriminating activity [i.e., y(t)] for each condition separately, we applied participant-specific spatial weights from the time window of peak discrimination to an extended stimulus-locked time window and separately for social and nonsocial trials. This approach revealed a gradual build-up of activity akin to a process of EA in both social and nonsocial trials (Fig. 4*c*, top, Social; bottom, Nonsocial). Similar to the classifier performance, the neural activity began to rise around 400 ms after stimulus presentation in both the social and nonsocial trials, with the build-up rate being proportional to the amount of decision difficulty. Note that the build-up rate from medium difficulty trials was situated between the two extreme conditions used to originally train the classifier, thereby establishing a fully parametric effect across the three levels of decision difficulty (*F*_(2,90)_ = 16.88, *p* < 0.001 for the social condition, *F*_(2,90)_ = 26.76, *p* < 0.001 for the nonsocial condition, *post hoc* paired *t* tests, all *p* < 0.001).

We finally extracted participant-specific EA onset times, that is the time point at which the discriminating activity began to rise monotonically after an initial dip in the data following any early evoked responses present in the data (Fig. 4*c*, insets). Because of the interindividual variability in these onset times, we predicted that re-aligning the relevant signals to the participant-specific EA onset times should reveal a more pronounced depiction of the underlying process of EA at the population level, which was indeed the case (Fig. 4*d*, top, Social; bottom, Nonsocial).

### Linking neural signatures of evidence accumulation to behavior

To further establish that our EEG signals reflect the process of EA leading up to the decision we performed two additional analyses to link these signals with our participants' behavioral performance. First, we expected the build-up rate of the EEG EA signals to correlate with drift rate estimates obtained from a sequential sampling model (SSM; Polanía et al., 2014; Pisauro et al., 2017) fit on participants' fraction of “Play” choices and RTs, which was indeed the case (Fig. 3; Social, Fraction “Play” Choice: *r* = 0.945, *t*_(154)_ = 36.464, two-sided *p* < 0.001, RT: *r* = 0.754; *t*_(154)_ = 15.154, two-sided *p* < 0.001; Nonsocial, Fraction “Play” Choice: *r* = 0.968, *t*_(154)_ = 94.196, two-sided *p* < 0.001, RT: *r* = 0.765; *t*_(154)_ = 14.461, two-sided *p* < 0.001).

In the SSM, we set the decision thresholds for “Play” and “Keep” choices to +1 and –1, respectively (see Materials and Methods for details). Correspondingly, positive (negative) drift rates are associated with reward probability levels favoring a “Play” (“Keep”) choice. To align the EEG build-up rates [i.e., linear slopes of y(t); see Materials and Methods] with this convention, we flipped the sign of the EEG slopes in the two reward probability levels which support “Keep” choices [i.e., P(payoff|play)={0−0.2,0.2−0.4}]. As expected, we observed robust correlations between the slopes of the EEG and drift rates in the model, across both social (Fig. 5*a*; *r* = 0.653, *p* < 0.001) and nonsocial trials (Fig. 5*b*; *r* = 0.709, *p* < 0.001). To exclude the possibility that our EEG slopes were reflective of changes in the remaining parameters, we also conducted correlations for the other (nonfixed) parameters in our model. As these parameters do not include modulations based on reward probability, we correlated the estimates with the mean EEG slopes per participant and found no significant correlations (all *p*-values > 0.05).

To further validate the association between the EEG EA and trial-wise changes in performance (rather than mean drift rates from the model as in the analysis above), we ran a logistic regression analysis (Eq. 8) to directly predict the probability of playing on individual trials based on the trial-by-trial slope estimates from the EEG EA signals. As expected, we found that EEG slopes were a significant predictor of the eventual probability of playing for both the social (Fig. 5*c*; *t*_(30)_ = 7.582, two-sided *p* < 0.001) and nonsocial trials (Fig. 5*d*; *t*_(30)_ = 8.173, two-sided *p* < 0.001).

We also asked whether including these trial-wise changes in the EEG EA slopes offers additional explanatory power into choice behavior, beyond what could be inferred using simple task-derived predictors such as reward probability (Eq. 9). We found that the patterns of results seen in our original behavioral analyses were still present in this regression analysis [i.e., that the P(Play) increased with reward probability], but that the deviance scores were lower for the combined analysis than the simple regressions suggesting that the addition of the neural predictor explained more of the variability in the data. Although the differences between the combined regression and the one relying on the task-derived reward probability predictor were small, they were significant for both domains (social: *t*_(30)_ = −4.924, *p* < 0.001; nonsocial: *t*_(30)_ = −6.515, *p* < 0.001).

Finally, we also tested the possibility that trial-by-trial modulations of the EEG slopes may have arisen merely because of fluctuations in attention (as it waxes and wanes in the course of the experiment). Specifically, we ran a linear serial autoregression model predicting the EEG-derived EA slope in the current trial from the slopes from the previous four trials, individually for all participants. We found that on average this only accounted for a very small portion of the overall variance in the EEG slopes (Social: *R*^2^ = 0.02, Nonsocial: *R*^2^ = 0.019), demonstrating the absence of a serial autocorrelation in slopes across neighboring trials.

### EEG-informed fMRI of evidence accumulation

Our EEG analysis demonstrated that both the social and nonsocial choices display comparable EA dynamics, potentially suggestive of common underlying neural generators. Here, we aimed to identify candidate regions involved in the process of EA for social and nonsocial contexts by using the endogenous trial-by-trial variability in the slope of EA (Fig. 6*a*). Crucially, trials with lower EA rates that require longer integration times to reach the decision boundary should have larger areas (energy) under the accumulation curve (Basten et al., 2010; Hare et al., 2011; Liu and Pleskac, 2011). Correspondingly, we hypothesize that candidate accumulator regions should appear to be more hemodynamically active in trials with longer compared with shorter integration times (Fig. 6*b*). This, in turn, should be consistent with a negative relationship between our EEG-informed EA slope predictor and the BOLD response in the relevant brain areas (Hare et al., 2011; Liu and Pleskac, 2011; Mulder et al., 2014).

Consistent with a domain-general EA neural architecture, we identified a region of the posterior-medial frontal cortex (pMFC) correlating negatively with the trial-by-trial EA slope in our EEG-informed predictor (Fig. 6*c*) in both social (*Z*(max) = 3.49, MNI [0, −16, 56]) and nonsocial contexts (*Z*(max) = 4.39, MNI [8, 8, 44]). We note that in nonsocial choices the cluster extended more anterior compared with social choices, suggestive of a potential gradient organization within the pMFC associated with EA. We found no domain-specific activations surviving in the direct contrast between social and nonsocial contexts for the EA slope predictor.

To further establish the role of the pMFC in EA across social and nonsocial contexts, we aimed to demonstrate whether its activity exhibits a task-dependent coupling with brain regions encoding the relevant decision evidence and the extent to which this coupling arises from domain-general or domain-specific neural representations. To this end, we ran separate psychophysiological interaction (PPI) analyses for each of the social and nonsocial trials, using the context-specific pMFC clusters as seed and the trial-wise task difficulty as the psychological predictor (see Materials and Methods for more details). We hypothesized that the relevant coupling with pMFC should be negative, as easier trials decrease integration times and correspondingly the overall integrated activity (that is, area under the accumulation curve; Fig. 6*b*).

**Figure 7.**
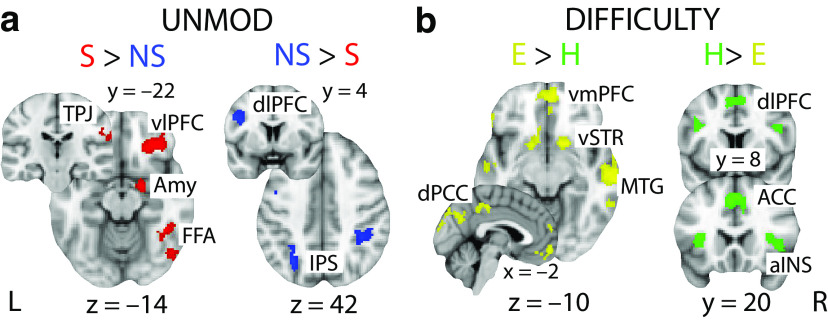
Domain-specific and task difficulty fMRI activations. ***a***, Activations showing greater BOLD response for social (S) than nonsocial (NS) trials (red) and those exhibiting higher response for nonsocial compared with social trials (blue). These activations arise from the contrast of the two unmodulated regressors (UNMOD) in Figure 6*a*. ***b***, Activations showing greater BOLD response for easy (E) than hard (H) trials (yellow) and those exhibiting higher response for hard compared with easy trials (green). These activations arise from the conjunction of the two task difficulty regressors (DIFF) for social and nonsocial trials in Figure 6*a*. All clusters represent mixed-effects activations that survived |Z|>2.57 and that were cluster-corrected (*p* < 0.05) using a resampling procedure with a minimum cluster size of 88 voxels (see Materials and Methods). vlPFC: ventrolateral prefrontal cortex; Amy: amygdala; FFA: fusiform face area; TPJ: temporoparietal junction; IPS: intraparietal sulcus; vmPFC: ventromedial prefrontal cortex; vSTR: ventral striatum; MTG: medial temporal gyrus; dPCC: dorsal posterior cingulate cortex; dlPFC: dorsolateral prefrontal cortex; ACC: anterior cingulate cortex; aINS: anterior insula.

The PPI analyses from both social and nonsocial contexts revealed significant negative coupling (by task difficulty during the decision phase) between the pMFC and regions of the human valuation system. More specifically, we observed activations in posterior cingulate cortex (PCC; Social *Z*(max) = 3.54, MNI [2, −70, 24]; Nonsocial *Z*(max) = 3.88, MNI [−4, −66, 22]) as well as in dorso- (Social *Z*(max) = 3.5, MNI [−4, 48, 26]; Nonsocial *Z*(max) = 3.39, MNI [−4, 52, 24]) and ventro-medial prefrontal cortex (dmPFC/vmPFC; Fig. 7, Social *Z*(max) = 3.19, MNI [−2, 52, 2]; Nonsocial *Z*(max) = 3.74, MNI [−8, 62, 0]), consistent with recent resting-state connectivity reports showing negative BOLD correlations between these regions and the pMFC (Neubert et al., 2015). Intriguingly, these areas have repeatedly been implicated in encoding a “common neural currency” of abstract value signals used in the process of EA (Rangel and Hare, 2010; Pearson et al., 2014; Piva et al., 2019). As with the EA clusters, we found that in the nonsocial context, activations were situated more anterior relative to the social context, consistent with previous reports of value gradients within the medial prefrontal cortex (Chib et al., 2009; D.V. Smith et al., 2010; Clithero and Rangel, 2014). Taken together, our findings provide compelling evidence that relevant decision evidence is converted into a “common currency” along the medial wall of the human brain and subsequently integrated for the decision in the pMFC. We found no domain-specific activations surviving in the direct contrast between social and nonsocial contexts for the PPI predictor.

### Domain-specific and task difficulty neural representations

To identify brain areas processing domain-specific representations (e.g., areas encoding the initial evidence for each of the two contexts before conversion to a “common currency”) we examined the contrast between the unmodulated predictors for the social versus nonsocial trials (Fig. 6*a*). We identified a distributed set of regions activating stronger for social compared with nonsocial trials, in the right fusiform gyrus (*Z*(max) = 5.18, MNI [40, −48, −16]), right amygdala (*Z*(max) = 4.48, MNI [18, −2, −16]) and right ventrolateral prefrontal cortex (*Z*(max) = 5.42, MNI [30, 34, −14]), collectively referred to as the “face network” (Skelly and Decety, 2012), as well as in the right temporoparietal junction (TPJ; *Z*(max) = 3.39, MNI [64, −42, 26]; Fig. 7*a*). These findings are consistent with the processing of facial characteristics required for mentalizing and inferring the opponent's intentions in the social trials, respectively (Cerniglia et al., 2019). In contrast, nonsocial trials exhibited increased activation patterns relative to social trials in areas of the lateral intraparietal cortex bilaterally (left *Z*(max) = 4.26, MNI [−28, −62, 46]; right *Z*(max) = 3.63, MNI [38, −42, 42]) as well as in the left dorsolateral preforntal cortex (dlPFC; *Z*(max) = 4.12, MNI [−38, 0, 32]; Fig. 7*a*), which have both been implicated in encoding risk and reward probabilities in nonsocial contexts (Daw et al., 2006; B.W. Smith et al., 2009; Burke and Tobler, 2011). We note that we view these results as further evidence that the social manipulation in our design was successful since we would not expect to see such differences in the neural data across contexts if the faces in social trials were simply used as proxies for the relevant reward probabilities (i.e., as in nonsocial trials).

Task difficulty in our task was reflective of the reward probability associated with a “Play” choice. We, therefore, expected the task difficulty predictor to correlate both positively (i.e., easy > difficult) with areas known to encode the value of a given choice and negatively (i.e., difficult > easy) with regions of the human attentional network encoding overall task demands. Across both social and nonsocial contexts, we found positive correlations with regions of the human valuation system such as the ventromedial prefrontal cortex (vmPFC; *Z*(max) = 3.75, MNI [6, 54, −8]), ventral striatum (left *Z*(max) = 3.54, MNI [−14, 8, −12]; right *Z*(max) = 3.88, MNI [16, 10, −10]) and the posterior cingulate cortex (*Z*(max) = 3.55, MNI [−2, −28, 46]; Clithero and Rangel, 2014; Domenech et al., 2018; Fig. 7*b*). Consistent with previous reports (Philiastides and Sajda, 2007; Grinband et al., 2008; Monosov, 2017), we also found negative correlations with regions encoding uncertainty and attentional control such as the anterior cingulate cortex (*Z*(max) = 5.09, MNI [−2, 16; 48]), lateral prefrontal cortex (left *Z*(max) = 4.11, MNI [−38, 8, 24]; right *Z*(max) = 3.34, MNI [44, 8, 24]) and anterior insula in both social and nonsocial contexts (Fig. 7*b*). We note, that though these results are consistent with a long body of previous work, they are nonetheless important to report here as further validation of the choice of our fMRI analysis design, which offered a reliable account of all relevant experimental manipulations.

## Discussion

The marriage of social and nonsocial forms of uncertainty into a comprehensive theory of decision-making promises to significantly improve our understanding of human behavior. Unlike previous examinations of social and nonsocial decisions across separate experiments or cohorts (Krajbich et al., 2015; Tarantola et al., 2017), here we enabled direct comparisons between domains by embedding both decision types in the context of an economic game in which task difficulty varied along the same scale of reward probability across social and nonsocial stimuli. Moreover, we extended previous reports that focused on group decisions (Suzuki et al., 2015; Park et al., 2019) or self-versus-other considerations (A. Harris et al., 2018) by examining choices in which the social information is carried by a social cue (i.e., facial features).

To this end, we examined whether the “common currency” framework, a view, which posits that a common neural architecture encodes an aggregate value of all factors guiding each of the social and nonsocial choices (Ruff and Fehr, 2014), extends beyond value encoding to include the process of integrating the relevant value signals for the decision. Indeed, we found that domain-specific perceptual information associated with each domain is likely converted into a common neural currency in regions of the human valuation system before being integrated for the decision in posterior-medial frontal cortex, thus expanding the scope of the “common currency” framework to include the EA stages of the decision process.

More specifically, modeling of choice-RT using a simple sequential sampling model was suggestive of comparable accumulation-to-bound dynamics across social and nonsocial contexts. Correspondingly, we identified ramp-like activity in the electrophysiological signal with a well-demarcated centroparietal scalp profile that was virtually identical across the two contexts. We further demonstrated that trial-by-trial variability in the slope of this accumulating activity was highly predictive of choice behavior. We capitalized on this endogenous trial-by-trial variability to deploy an EEG-informed fMRI analysis, to implicate a region of the pMFC in the process of EA across both social and nonsocial contexts. These findings suggest that both decision types are likely to rely on the same implementational and algorithmic process (Lockwood et al., 2017), consistent with previous reports in value-based (Gluth et al., 2012; Polanía et al., 2014; Pisauro et al., 2017), perceptual (Drugowitsch et al., 2012; Kelly and O'Connell, 2013; Gherman and Philiastides, 2015), and even memory-based decisions (van Vugt et al., 2019).

The pMFC cluster we identified here lies on the medial surface of the juxtapositional lobule cortex in a region commonly referred to as the SMA and extending into portions of the adjacent pre-SMA, bilaterally. Both areas have traditionally been linked to preparing voluntary actions (Nachev et al., 2008; Kim et al., 2010) but have also been assigned other functional roles, including decision boundary adjustments in the context of accumulation-to-bound models (Forstmann et al., 2008; Ivanoff et al., 2008). Our results suggest that the role of the pMFC extends beyond mere decision boundary adjustments and involves instead the encoding of the full temporal dynamics of the process of EA, a process that might be mediated by an increased tendency to select the appropriate motor response. Correspondingly, our results support the rapidly emerging view that, at least under conditions of increased urgency to make a choice, decisions are embodied in the same sensorimotor areas guiding the actions used to express that choice. This interpretation is consistent with past neuroimage findings (Donner et al., 2009; Filimon et al., 2013) and recent mechanistic accounts ascribing an active “motor accumulation” role to (pre)motor structures (Steinemann et al., 2018; Verdonck et al., 2021).

To further probe the implementational level of social and nonsocial decisions, we performed a psychophysiological interaction (PPI) analysis. This analysis allowed us to investigate the functional coupling of the pMFC with other brain regions to further probe the extent of the “common currency” account introduced above. Unlike some recent accounts, which have argued that social and nonsocial values are computed across different networks (Ugazio et al., 2022), we found that pMFC activity exhibited task-dependent coupling (i.e., as the process of EA unfolds) with regions of the human valuation system in the dmPFC, vmPFC, and PCC (Rangel and Hare, 2010; Pearson et al., 2014; Piva et al., 2019). This coupling was present in both social and nonsocial choices, consistent with the relevant decision evidence being converted into a “common currency” before being used in the process of EA to drive the final commitment to choice.

We speculate, that this discrepancy could potentially be attributed to differences in the types of social choices (i.e., choices based on a social cue as utilized here versus the moral choices used by Ugazio et al., 2022). Specifically, when considering moral versus social choices, Ugazio et al. (2022) argue that moral choices are influenced by the social context to a much lesser extent than social choices and highlight the fact that the ability to differentiate between social and moral scenarios emerges from an early age (Smetana, 1981; Smetana et al., 2013) potentially because of the fact that these two decision types employ separate networks (Moll et al., 2008).

Similarly, differences in social versus nonsocial processing based on self-versus-other decisions (A. Harris et al., 2018) can be attributed to the difference in benefactors (as the decision on behalf of someone else would likely be linked to higher uncertainty). In addition, deciding on someone else's behalf could be linked to a two-stage process, where the outcome of an action is considered initially in accordance with one's own views and then re-adjusted to fit someone else's. Therefore, by closely matching the social and nonsocial conditions in the current experiment, we may be in a better position to observe the similarities between the processing of social and nonsocial decisions.

While pMFC activity covaried systematically with areas of the human reward network, we nonetheless observed some degree of spatial dissociation across contexts along an anterior/posterior axis within these areas, consistent with recent reports from human and animal work advocating for a gradient-based organization along the medial wall of the brain (Kolling et al., 2021). Specifically, in the social context the relevant clusters were situated within the most posterior sections of the medial prefrontal cortex (Ferrari et al., 2016; Lieberman et al., 2019) whereas those of the nonsocial context occupied sections of the most anterior portions of this region (Chib et al., 2009; D.V. Smith et al., 2010; Clithero and Rangel, 2014). Interestingly, this organization seems to be preserved along the cascade of constituent processes, from the relevant value representation to the process of EA.

The “common currency” account also postulates that before embedding within a domain-general valuation system, social and nonsocial choices might give rise to domain-specific early representations (Lim et al., 2013; Hutcherson et al., 2015). Here, we observed that social choices were associated with increased activity in the FFA, the amygdala and vlPFC, with a right lateralization, consistent with the well-known “face network,” which is crucial for face identification and affective processing of faces (Vuilleumier et al., 2004; Garvert et al., 2014). Similarly, we observed increased activation in the right TPJ, a region implicated in social cognition and various types of mentalizing relevant for extracting semantic meaning (value) from an opponent's face identity (Van Overwalle, 2009). While a potential shortcoming of our experiment is that we did not explicitly ask our participants whether they believed our cover story (i.e., that the face stimuli belonged to people who have previously taken part in a Trust game), these uniquely social activations demonstrate that participants were relying on relevant domain-specific information to guide their choices.

Conversely, the nonsocial condition was uniquely linked to activity in the lateral intraparietal cortex which has been linked to the encoding of pure reward probabilities both in primates (Sugrue et al., 2004; Burke and Tobler, 2011) and humans (Daw et al., 2006; Wu et al., 2015), suggesting that these choices were guided by the consideration of the likelihood of receiving a reward as presented explicitly during the task. Collectively, these representations are indeed evidence of task-relevant information being broadcasted directly to the prefrontal cortex (Heekeren et al., 2004; Philiastides and Sajda, 2007; Pessoa and Adolphs, 2010) from domain-specific brain regions (Lamichhane and Dhamala, 2015), with these feed-forward projections being among the main pathways connecting to higher-level prefrontal areas (Yeterian et al., 2012; Lamichhane and Dhamala, 2015).

More importantly, the proposed “common currency” account is in line with our ability to make choices based on disparate options that ultimately require a common point of convergence in the neural code. Consistent with this idea there has been empirical evidence suggesting that the medial prefrontal cortex has the capacity to both encode the subjective values of individual options as well as re-scale these values into a common neural activity space (Chow et al., 2009; Levy and Glimcher, 2011). In turn, this mechanism could enable direct comparisons between different choice alternatives and thereby guide decision-making in a domain-independent manner.

In conclusion, our results offer compelling new evidence that social and nonsocial choices share common neural underpinnings, whereupon domain-specific information is converted into a “common currency” in domain-general valuation areas before being accumulated for the decision in medial frontal cortex. Similarly, our multimodal research approach, including the fusion of EEG and fMRI, offers news opportunities for a more targeted characterization of the mechanistic principles and the neural systems involved in human decision-making under different forms of uncertainty. As optimal decision-making is at the heart of strategic planning, providing a mechanistic account of decision-making under risk and uncertainty could have wider, long-term, socioeconomic impact and facilitate an improved understanding of maladaptive choice behavior.
